# Clinical outcomes and quality of life in hemodialysis diabetic patients versus non-diabetics

**DOI:** 10.15171/jnp.2017.14

**Published:** 2016-12-14

**Authors:** Tayebeh Soleymanian, Zeinab Kokabeh, Rozita Ramaghi, Alireza Mahjoub, Hassan Argani

**Affiliations:** ^1^Nephrology Research Center, Shariati Hospital, Tehran University of Medical Sciences, Tehran, Iran; ^2^Nephrology Research Center, Shariati Hospital, Tehran University of Medical Sciences-International Branch, Tehran, Iran; ^3^Urology and Nephrology Research Center, Modares Hospital, Shahid Beheshti University of Medical Sciences, Tehran, Iran

**Keywords:** Hemodialysis, Patient outcomes, Diabetes mellitus, Quality of life, Cardiovascular disease

## Abstract

**Background:**

Diabetes is the leading cause of end stage renal disease (ESRD) worldwide.

**Objectives:**

We compared the clinical outcomes in diabetic patients on hemodialysis (HD) with non-diabetics.

**Patients and Methods:**

Adult maintenance HD patients (N= 532) from 9 HD facilities were enrolled to this prospective cohort study in September 2012. Causes of death, hospitalization, and HD exit were recorded in a median 28 months follow up period.

**Results:**

Forty-one percent of patients were diabetic. Diabetic patients compared to non-diabetics had significantly higher age (62.2 ± 11.2 versus 53.1 ± 16.7 years), lower dialysis duration (median: 23 versus 30 months), more cardiovascular comorbidities (64% versus 28%) , higher C-reactive protein (CRP) levels (median: 3.80 versus 2.25 mg/L), lower serum albumin (3.86 ± 0.35 versus 3.93 ± 0.35 g/dL), lower intact parathyroid hormone (iPTH) (median: 272 versus 374 ρg/mL), higher serum triglyceride (167 ± 91 versus 139 ± 67 mg/dL) and low density lipoprotein (LDL) (82.5 ± 24.5 versus 77.5 ± 23.8 mg/dL), and worse short form health survey (SF36) score (45.7 ± 20.9 versus 52.7 ± 20.5). Annual admission rate was higher in diabetics (median: 0.86 versus 0.43) and diabetic foot involved 16% of their admissions. Transplantation rate was 4 and 9 per 100 patient years in diabetics and non-diabetics, respectively. Death rate was two folds higher in diabetics (24 versus 12 per 100 patient years). Cardiovascular diseases ( ± infections/other causes) comprised 80.5% of death in diabetics and 54.5% in non-diabetics. In Cox regression proportional hazard multivariate analysis, hazard risk of death in diabetics was 1.9 times higher than non-diabetics.

**Conclusions:**

Clinical outcomes and health related quality of life (HRQOL) are much worse in diabetic compared to non-diabetic HD patients mainly due to more frequent of cardiovascular diseases (CVDs).

**Implication for health policy/practice/research/medical education:**

Diabetic nephropathy is the leading cause of ESRD worldwide. We tried to define patients characteristics, common causes of death and its’ risk factors in diabetic HD patients in order to delineate intervensions which could potentially help to improve patients’ quality of life and survival.

## 1. Background

Diabetes is the most common cause of end stage renal disease (ESRD) in the United States and most other developed and emerging countries (1). It accounts for almost 45% cause of ESRD in the United States (2,3). However, recently the incidence of diabetic nephropathy requiring dialysis has stabilized or decreased in some developed countries owing to widespread practice of severe renoprotective measures in diabetic patients (3,4).

Outcome of diabetic patients on maintenance dialysis is worse than non-diabetics with ESRD cause of hypertension or glomerular diseases, with marginal improvement in recent reports (5,6). It is the best in young diabetic patients with no cardiovascular diseases (CVDs) (7).

## 2. Objectives

In this study, we tried to determine the characteristics, quality of life, comorbidities and death rate of diabetic patients on maintenance hemodialysis (HD) in nine facilities. We also compared the characteristics and outcome of HD diabetic patients with non-diabetics.

## 3. Patients and Methods

### 3.1. Study population

In this study, 532 maintenance HD patients from nine facilities were recruited in September 2012. The enrolled facilities which their authorities and medical staff signed to collaborate were from the high, intermediate, and low socioeconomic regions of Tehran. All patients had to be at least 18 years old and receiving outpatient HD at least for two weeks. A comprehensive questionnaire comprising all demographic, clinical and laboratory information was prepared. At least two to three constitutive laboratory data at study start were recorded in the questionnaire (their mean was used for analysis). Then, patients were followed up until February 2015. During follow up period (median of 28 months; minimum of 0.5 and maximum of 30 months) causes of hospitalizations and exit from HD including death was recorded. The last follow up time was the last visit or whenever patients left HD because of renal recovery, transfer to peritoneal dialysis (PD) or undergoing transplantation (one month after PD transfer or transplantation). Patients who transferred to a different facility were followed up there. Patients were considered to have coronary artery disease (CAD) if they underwent coronary artery bypass graft (CABG); percutaneous coronary intervention (PCI), or on medical therapy because of CAD diagnosis by coronary angiography, dobutamine stress echocardiography, or stress myocardial perfusion imaging. Diagnosis of congestive heart failure was made by echocardiographic criteria.

We also evaluated the health related quality of life (HRQOL) using the 36-item short form health survey (SF36) that measures SF36 score and eight subscales and two dimensions of physical component summary (PCS) and mental component summary (MCS) in accordance with a scoring algorithm (8,9).

### 3.2. Ethical issues

1) The research followed the tenets of the Declaration of Helsinki; 2) written informed consent was obtained, and they were free to leave the study at any time; and 3) the research was approved by the ethical committee of Shahid Beheshti University of Medical Sciences (Grant ≠ 910825-12).

### 3.3. Statistical analysis

Demographic characteristics and laboratory data of the patients are summarized using percentage of the total and means ( ± standard deviation, SD) or medians (interquartile range) as appropriate. Mean values of the last two to three laboratory results at the study start for each patient were used in the analysis. Categorical variables were compared using chi-square or Fisher’s exact tests and continuous variables were compared using *t* test and analysis of variance (ANOVA) or Mann–Whitney U and Kruskal-Wallis as appropriate. Cox proportional hazard models were used for hazard ratios (HR) of death while controlling for the relevant covariates. Follow up time for each patient was the time of event (death) or censoring (recovery, PD, transplantation or last visit), whichever developed first. Death in the first month of transfer to PD or transplantation was included. Unadjusted and incremental levels of multivariate adjustment were used as case-mix variables (age, sex and HD vintage), nutrition variables (serum albumin, creatinine, pre-dialysis blood urea nitrogen [BUN], hemoglobin, transferrin, body mass index [BMI]), bone mineral variables (calcium, phosphorus, and iPTH), single pool Kt/V, vascular access, and diabetes. Patient survival for diabetics and non-diabetic patients was estimated by the Kaplan–Meier method. Predictors of mortality in diabetics were illustrated by Kaplan-Meier curve. The data analysis was performed using SPSS version 19 (SPSS Inc., Chicago, IL). Significant level was considered as *P* < 0.05.

## 4. Results

The mean age of patients (n = 532) was 56 ± 15.4 years which 57% (n = 302) of them were men and 41% (n = 219) were diabetic. The mean duration on dialysis was 44.6 ± 49.1 (median: 25, IQR: 11-66) months. Forty-one patients (7.5%) were on dialysis for ≤3 months (incident patients) which 17 of them (41.5%) were diabetics.

In univariate analysis (Table 1), diabetic patients in comparison with non-diabetics were significantly older (62.2 ± 11.2 versus 53.1 ± 16.7 years), with lower dialysis duration (median: 23 versus 30 months), and higher CRP levels (median: 3.80 versus 2.25 mg/L). Although diabetic patients revealed significantly higher BMI (25.6 ± 4.6 versus 23.5 ± 4.3 kg/m^2^) but serum albumin (3.86 ± 0.35 versus 3.93 ± 0.35 g/dL) and creatinine (7.6 ± 2.4 versus 9.3 ± 2.7 mg/dL) were significantly lower in diabetics. Proportion of overweight plus obese patients was higher in diabetic patients (51% versus 33%). In terms of lipid profile, diabetic patients had significantly higher serum triglyceride (167 ± 91 versus 139 ± 67 mg/dL) and low density lipoprotein (LDL) (82.5 ± 24.5 versus 77.5 ± 23.8 mg/dL) but lower high density lipoprotein (HDL) level (35.9 ± 7.5 versus 37.7 ± 9.2 mg/dL). Regarding metabolic bone profile, diabetics had significantly lower iPTH (median: 272 versus 374 ρg/mL) and serum phosphorus level (5.2 ± 1.2 versus 5.6 ± 1.2 mg/dL). Dialysis adequacy (single-pool Kt/V) was significantly lower in diabetic patients (1.27 ± 0.19 versus 1.33 ± 0.21).

### 4.1 .Vascular access

Various vascular accesses were evenly distributed in both diabetic and non-diabetic patients, though there was a trend for more catheter use in diabetic patients (22% versus 16%, *P* = 0.09) (Table 1).

There was a significant difference in vascular access type usage in diabetic patients based on sex, such that in male diabetics use of arteriovenous fistula (AVF), arteriovenous graft (AVG), and tunneled central venous catheter (CVC) were respectively 82% (n = 90), 2% (n = 2), 17% (n = 19) while these numbers for females were 62% (n = 63), 11% (n = 11), and 27% (n = 28), respectively (*P* = 0.002). This *P* value for use of CVC versus non-CVC access according to sex in diabetics was 0.05. Mean age for use of AVF, AVG, and tunneled CVC in diabetics were respectively 61.5 ± 10.4, 68.3 ± 10.9, and 62.7 ± 13.8 years (*P* = 0.10).

### 4.2. Health related quality of life

Patients with diabetes had significantly worse quality of life (Table 1). SF36 score in diabetics was 45.7 ± 20.9 versus 52.7 ± 20.5 in non-diabetics. All SF36 subscales except social functioning and bodily pain were significantly inferior in diabetes. Also, both physical and mental component summaries were worse in diabetic patients. Unadjusted hazard ratio of death for SF36 score was 1.19 (95% CI: 1.07-1.32; *P* = 0.002) which became progressively weaker after adjustment for demographic data (age, sex, and dialysis vintage) and then added serum albumin and finally included CAD; with numbers of 1.16 (95% CI: 1.04-1.28; *P* = 0.01), 1.11 (95% CI: 0.99-1.24; *P* = 0.07), 1.07 (95% CI: 0.94-1.18; *P* = 0.28), respectively. Unadjusted HR of death for PCS was 1.17 (95% CI: 1.05-1.28; *P* = 0.005) and became 1.14 (95% CI: 1.02-1.25; *P* = 0.02) after adjustment for demographic data. Likewise, unadjusted HR of death for MCS was 1.11 (95% CI: 0.999-1.23; *P* = 0.06).

**Table 1:** Comparison of demographic and laboratory data between non-diabetic and diabetic HD patients

**Characteristics**	**Non-diabetes** (n= 313; 59%)	**Diabetes** (n= 219; 41% )	P value
Demographic data:			
Sex (male)%	187 (60%)	115 (52.5%)	0.09
Age (y)	53.1 ± 16.7	62.2 ± 11.2	<0.001
HD vintage^a^ (mon)	30 (IQR: 11-74)	23 (10-52)	0.01
BMI (kg/m^2^)			<0.001
≤ 18.5	18 (5.8%)	7 (3.2%)	
> 18.5- 25	192 (61.3%)	100 (45.6%)	
> 25- 30	84 (26.8%)	77 (35.2%)	
> 30	19 (6.1%)	35 (16%)	
Vascular access type^b^			0.19
AVF	231 (76%)	153 (72%)	
AVG	24 (8%)	13 (6%)	
Tunneled CVC	49 (16%)	47 (*22*%)	
Laboratory data (serum):			
Albumin (g/dL)	3.93 ± 0.35	3.86 ± 0.35	<0.001
CRP^a^ (mg/L)	2.25 (IQR: 0.90-5.13)	3.80 (1.50-8.30)	0.002
Hemoglobin (g/dL)	10.4 ± 1.6	10.6 ± 1.4	0.06
Creatinine (mg/dL)	9.3 ± 2.7	7.6 ± 2.4	<0.001
Pre-dialysis BUN (mg/dL)	56.5 ± 13.7	56.2 ± 13.1	0.86
Single-pool Kt/V	1.33 ± 0.21	1.27 ± 0.19	0.001
Triglyceride (mg/dL)	139 ± 67	167 ± 91	<0.001
Cholesterol (mg/dL)	147 ± 37	152 ± 35	0.12
LDL (mg/dL)	77.5 ± 23.8	82.5 ± 24.5	0.045
HDL (mg/dL)	37.7 ± 9.2	35.9 ± 7.5	0.032
Potassium (meq/L)	5.1 ± 0.6	5.2 ± 0.6	0.07
Calcium (mg/dL)	8.8 ± 0.7	8.8 ± 0.6	0.97
Phosphorus (mg/dL)	5.6 ± 1.2	5.2 ± 1.2	0.005
Alkaline phosphtase^a^ (IU/L)	324 (IQR: 199-449)	283 (IQR: 213-365)	0.08
iPTH^a^(pg/mL)	374 (IQR: 173-707)	272 (150-420)	<0.001
Iron (µg/dL)	69 ± 35	66 ± 39	0.53
Transferrin (µg/dL)	248 ± 59	253 ± 58	0.41
Ferritin (ng/mL)	446 ± 295	407 ± 247	0.11
Quality of life			
SF36 score	52.7 ± 20.5	45.7 ± 20.9	<0.001
Physical functioning	52.8 ± 29.5	40.2 ± 31.3	<0.001
Role-physical	52.4 ± 30.7	43.2 ± 30.7	0.003
Bodily pain	61.5 ± 31.2	60.4 ± 31.9	0.73
General health	51.7 ± 24.1	46.9 ± 21.2	0.03
Vitality	50.6 ± 26.6	44.7 ± 25.9	0.02
Social functioning	52.7 ± 30.4	47.9 ± 29.2	0.10
Role-emotional	60.1 ± 35.8	50.5 ± 35.1	0.008
Mental health	61.3 ± 25.5	56.1 ± 27.7	0.05
Physical component summary	53.8 ± 21.6	47.1 ± 21.8	0.004
Mental component summary	55.3 ± 22.2	49.3 ± 21.1	0.005

Abbreviations: BMI, body mass index; LDL, low density lipoprotein; HDL, high density lipoprotein; HD, hemodialysis; iPTH, intact parathyroid hormone; SF36, short form health survey; BUN, blood urea nitrogen; AVF, arteriovenous fistula; AVG, arteriovenous graft; CRP, C-reactive protein. ^a^ Median and inter-quarter range; ^b^Non-tunneled CVC is not included.

### 4.3. Patient outcome

Annual admission number for diabetic patients was 0.86 (IQR: 0.43-1.29) and for non-diabetics was 0.43 (IQR: 0.00-0.85) (*P* < 0.001; Table 2). Diabetic foot frequency during study was 58% and diabetic foot involved 16% of the admissions (0.16 per patient year). Overall, patient follow up for diabetics was 4480 months (with admission numbers of 373 and death numbers of 91) and for non-diabetics was 6902 months (with admission numbers of 348 and death numbers of 70). Death happened in 42% of diabetic patients (24 per 100 patient years) and in 22.5% of non-diabetics (12 per 100 patient years). Rate of renal transplantation was 4 per 100 patient years in diabetics and 9 per 100 patient years in non-diabetic patients. Cardiovascular diseases ± infections/other causes comprised 80.5% of death in diabetics and 54.5% in non-diabetics. Infections ± CVD causes included 31.5% of death in diabetic patients and 17% of the non-diabetics. Rate of malignancy as a cause of death did not differ in two groups (7%).

**Table 2:** Comparison of cardiovascular comorbidities and clinical outcomes between non-diabetic and diabetic HD patients

**Characteristics**	**Non-diabetes** **(n= 313 )**	**Diabetes** **(n= 219 )**	**P value**
Cardiovascular comorbidities			
CAD			<0.001
None	225 (72%)	79 (36%)	
Medical therapy	53 (17%)	86 (39.5%)	
Stent	12 (4%)	16 (7%)	
CABG	23 (7%)	38 (17.5%)	
Congestive heart failure	17%	37%	<0.001
Cerebrovascular accident	8%	17%	0.003
Clinical peripheral vascular disease	0.94%	57%	<0.001
HD exit causes			<0.001
Still on HD	183 (58.5%)	111 (50%)	
Death	70 (22.5%)	91 (42%)	
Renal transplantation	53 (17%)	14 (6.5%)	
Peritoneal dialysis	5 (1.5%)	2 (1%)	
Renal function recovery	2 (0.5%)	1 (0.5%)	
Death causes			0.02
CVD	30 (43%)	37 (40.5%)	
Others+ CVD	2 (3%)	12 (13%)	
Infections + CVD	6 (8.5%)	25 (27%)	
Infections	6 (8.5%)	4 (4.5%)	
Malignancy	5 (7%)	6 (7%)	
Cachexia (±CVD/infection)	7 (10%)	3 (3.5%)	
Others	14 (20%) (two post-transplantation)	4 (4.5%) (one post-transplantation)	
Hospitalization rate			
Annual admission number	0.43 (0.00-0.85) 0.6 per patient-year	0.86 (0.43-1.29) 1 per patient-year	<0.001
Annual admission number (diabetic foot)	-	25/162 (16%)	

Abbreviations: CVD, cardiovascular diseases; CAD, coronary artery disease; CABG, coronary artery bypass graft; HD, hemodialysis.

**Figure 1:**
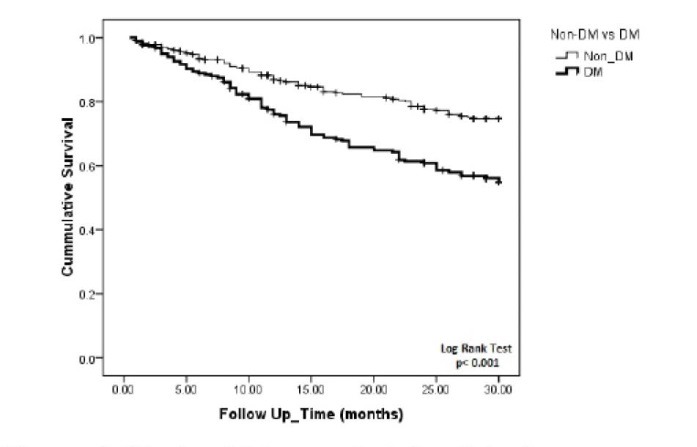
Kaplan-Meier survival for diabetic versus nondiabetic patients

We applied Cox regression proportional hazard analysis to ascertain variables which are predictive of overall mortality. After multivariate adjustment for case-mix, nutritional factors, bone mineral markers, single pool Kt/V and vascular access; diabetic patients still had 1.9 fold higher risk for death compared to non-diabetics (95% CI: 1.25-2.89; *P* = 0.003) (Figure 1). We decided that this higher mortality would be because of higher CVDs including CAD (64% versus 28%, *P* < 0.001), congestive heart failure (37% versus 17%, *P* < 0.001), cerebrovascular accident (17% versus 8%, *P* = 0.003), and clinical peripheral vascular disease (57% versus 0.94%, *P* < 0.001) in diabetic patients compared to non-diabetics. Therefore, we added cardiovascular variables in the Cox model and observed that diabetes was no longer independent predictors of death.

**Figure 2:**
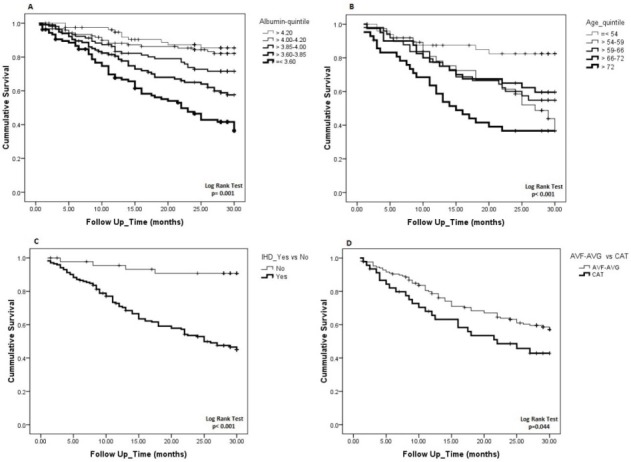
Kaplan-Meier survival based on independent predictors of mortality in diabetic HD patients. (A) Kaplan-Meier survival based on serum albumin-quintiles (≤3.60, >3.60-3.85, >3.85-4.00, >4.00-4.20, >4.20 g/dL; P = 0.001). (B) Kaplan- Meier survival based on age-quintiles (≤54, >54-59, >59-66, >66-72, >72 years; P < 0.001). (C) Kaplan-Meier survival based on ischemic heart disease (P < 0.001). (D) Kaplan-Meier survival based on vascular access (P = 0.044).

We also used Cox regression proportional hazard analysis to determine variables which are predictive of diabetics’ mortality. After multivariate adjustment for age, sex, HD vintage, serum albumin, CAD , Kt/V, access type and BMI; the independent predictors of mortality were; age (year) (HR: 1.02; 95% CI: 1.00-1.04, *P* = 0.05), serum albumin (g/dL) (HR: 0.18; 95% CI: 0.08-0.38, *P* ≤ 0.001), CAD (HR: 4.75; 95% CI: 1.67-13.51, *P* = 0.003), and a trend for catheter assess (HR: 1.70; 95% CI: 0.93-3.13, *P* = 0.08; Figure 2).

## 5. Discussion

In this multicenter study, we compared clinical outcome and its predictors in HD diabetic patients with non-diabetics recruited in 2012 and followed up for a median of 28 months. Forty- one percent of our patients were diabetics which indicated that diabetic nephropathy is a common cause of ESRD in our country. Prevalence of diabetic nephropathy in maintenance HD patients in DOPPS 4 (2011) countries reported to be in the range of 24% (Italy) to 54% (USA). However, the rate of new ESRD cases with diabetes has decreased in recent years in the United States (2).

Lower dialysis vintage in present study which is in consistent with report by Cano et al (10) represents the poor survival of diabetic patients. Indeed, because transplantation rate in diabetics was lower than non-diabetics we can conclude that increased death leads to lower dialysis duration in diabetics.

Whereas the number of overweight and obese patients was higher in diabetic patients (51% versus 33%), nutritional markers of serum creatinine and albumin was inferior in these patients. As a matter of fact, diabetic patients are confronted to malnutrition owing to low protein intake and greater catabolic state. As shown by other studies (10,11), higher BMI in these patients symbolizes an increment of fat portion because they actually have reduced lean body mass. Serum albumin is a marker of nutrition and strong predictor of mortality in HD patients (12).

Decreased appetite relevant to lower dialysis adequacy and uremia environment along with gastroparesis engender diabetics to reduced intake of protein (13). In addition, inflammation (higher CRP levels) induced by underlying comorbidities and infections; such as diabetic foot and infections related to higher usage of catheter access; through increase muscle catabolism and suppression of appetite contribute to malnutrition and cachexia (14,15). On the other hand, in patients with adequate dialysis, acute phase response is dominant cause of low serum albumin with higher mortality risk and the role of protein intake is minimal. Therefore, hypoalbuminemia should be considered as a marker of patients’ illness which needs interventions to improve patient survival (16,17).

Lipid disorders are common in diabetic patients and in present study we observed a higher LDL-C and triglyceride levels while HDL-C level was lower compared to non-diabetics. Lipid abnormalities as one of the traditional risk factor for atherosclerosis can somewhat explain further cardiovascular diseases in diabetic patients (18,19).

It has been demonstrated that low turnover metabolic bone disease is quite common in diabetic patients (20,21). We observed that both iPTH and serum phosphorus was lower in diabetic patients. There is some evidence that low turnover bone disease might be as a result of malnutrition, inflammation and raised oxidative stress; which leading to higher cardiovascular disease and mortality in dialysis patients (22,23). Also, our diabetic patients were older than non-diabetics which would aggravate low turnover bone disease in these patients.

In terms of vascular access, we recognized a trend for higher catheter utilization with resultant more infections in diabetic patients. There was a greater use of non-AVF access in diabetic women and a trend in older patients which are in accordance with other reports (24). Catheter usage with accompanying less dialysis adequacy and greater risk of infection and inflammation could potentially affect the survival of the HD patients (25). Furthermore, infections in the context of cardiovascular disease could potentially exacerbate the underlying CVD and expedite death of the HD patients. It is also established that inﬂammation intensifies cardiovascular risk and relevant mortality in HD patients (26).

Mortality of diabetic patients is still highest among HD patients (18). The poor survival is mainly connected to greater cardiovascular disease in these patients (27-29). We have shown that cardiovascular disease comprising CAD (64% versus 38%), congestive heart failure (37% versus 17%), and cerebrovascular accident (17% versus 8%) were much higher in diabetic compared to non-diabetic patients. Mortality rate in diabetes was two folds and cardiovascular diseases ( ± infections/other causes) comprised 80.5% of death compared to 54.5% in non-diabetics. Also, death owing to infections ( ± CVD) was quite more common in diabetics (31.5% versus 17%). However, proportion of malignancy as cause of death was the same in two groups (7%). Diabetic foot infection ( ± CVD) and catheter infection ( ± CVD) comprised 11% and 14% of the diabetics’ death, respectively. With progression of dialysis therapy the features of cardiac and vascular diseases accelerated and it has been reported that the frequency of diabetic foot became 2-5 folds higher in dialysis patients in contrast to pre-dialysis period (30,31). While, kidney transplantation is associated with better quality of life and increased patient survival, it is recommended that suitable diabetic patients proceeded to transplantation even preemptively, before progressive cardiovascular problems constrain the renal transplantation (32).

One of the causes of death in HD patients particularly in diabetics is withdrawal from dialysis (33), however this event was interestingly zero in present study.

In this study, the independent predictors of mortality in diabetic patients were older age, low serum albumin, CAD and a trend for catheter vascular access. These findings are in accordance with results of the most other studies (7,25,27,28).

We detected that health-related quality of life and both its’ components of physical and mental health were significantly inferior in diabetic patients compared to non-diabetics. Notably, health perception of the physical functioning, role physical and role emotional were the worst in dialyzed diabetic patients. HRQOL and its’ physical component predicted mortality of diabetic patients on HD. Not surprisingly, lower HRQOL indicated the more underlying comorbidities which independently and significantly affected outcome of HD patients with diabetes. Other studies have shown reduced health perceived physical aspects per se or both physical and mental aspects in HD diabetic patients (34,35). In the same way, most studies have reported that PCS is the predictor of mortality in HD diabetics while the minority has observed the further impact of MCS on patient outcome (34,36,37).

## 6. Conclusions

In summary, diabetic nephropathy is the leading cause of ESRD and it conveys a poor outcomes. The unfavorable prognosis is mostly due to higher frequency and more severe cardiovascular diseases which constantly deteriorated as the duration of dialysis elapsed. Poor quality of life is consequence of underlying cardiovascular disease as well. The detrimental effects of high glucose associated with poor lipid profile and inflammation contribute to growing atherosclerosis and progressive CVD. Therefore, prompt diagnosis and management of cardiac and vascular problems including diabetic foot complications in conjunction with timely kidney transplantation in eligible patients is strongly recommended. Additionally, proper handling of vascular access, treatment of infections, and CVD risk reduction is an invaluable deal.

## Strengths and limitations of the study

Present study introduced a comprehensive data covered nearly all patients’ characteristics, laboratory data and comorbidities from nine HD facilities, such that we were able to evaluate typical features of the patients and also estimate the predictors of clinical outcomes. However, we did not use time-average values for variables as some variables will be changed along the time course, although we utilized the average of at least two or three laboratory data at study entry. Other limitations are observational study, not considering the residual renal function, evaluating HRQOL at one time, and perhaps persistence of other residual confounders.

## Conflicts of interest

The authors declare no conflict of interest.

## Authors’ contribution

TS and HA conducted the research. ZK and RR and AM collected the data. TS analyzed the data. TS prepared the primary draft. HA edited the final draft. All authors signed the manuscript.

## Funding/support

The authors are grateful to the “Urology and Nephrology Research Center” of Shahid Beheshti University of Medical Sciences for approving the project (Grant ≠ 910825-12) and providing financial support.

## References

[1] Van Dijk PC, Jager KJ, Stengel B, Gronhagen-Riska C, Feest TG, Briggs JD (2005). Renal replacement therapy for diabetic end-stage renal disease: data from 10 registries in Europe (1991–2000). Kidney Int.

[2] Collins AJ, Foley RN, Herzog C, Chavers BM, Gilbertson D, Ishani A (2010). Excerpts from the US renal data system 2009 annual data report. Am J Kidney Dis.

[3] CDC (2010). Incidence of end-stage renal disease attributed to diabetes among persons with diagnosed diabetes---United States and Puerto Rico, 1996-2007Disease Control. MMWR Morb Mortal Wkly Rep.

[4] Sørensen V, Hansen P, Heaf J, Feldt-Rasmussen B (2006). Stabilized incidence of diabetic patients referred for renal replacement therapy in Denmark. Kidney Int.

[5] Wolfe RA, Gaylin DS, Port FK, Held PJ, Wood CL (1992). Using USRDS generated mortality tables to compare local ESRD mortality rates to national rates. Kidney Int.

[6] USRDS U (2005). Excerpts from the USRDS 2005 Annual Data Report. Am J Kidney Dis.

[7] Locatelli F, Pozzoni P, Del Vecchio L (2004). Renal replacement therapy in patients with diabetes and end-stage renal disease. J Am Soc Nephrol.

[8] Ware JE Jr, Sherbourne CD (1992). The MOS 36-item short-form health survey (SF-36): I Conceptual framework and item selection. Med Care.

[9] Ware JE, Kosinski M, Keller S. SF-36 physical and mental health summary scales: a user's manual: health Assessment Lab; 1994.

[10] Cano NJ, Roth H, Aparicio M, Azar R, Canaud B, Chauveau P (2002). Malnutrition in hemodialysis diabetic patients: evaluation and prognostic influence. Kidney Int.

[11] Spotti D, Librenti M, Melandri M, Slaviero G, Quartagno R, Vedani P (1993). Bioelectrical impedance in the evaluation of the nutritional status of hemodialyzed diabetic patients. Clin Nephrol.

[12] Iseki K, Kawazoe N, Fukiyama K (1993). Serum albumin is a strong predictor of death in chronic dialysis patients. Kidney Int.

[13] Owen Jr WF, Lew NL, Liu Y, Lowrie EG, Lazarus JM (1993). The urea reduction ratio and serum albumin concentration as predictors of mortality in patients undergoing hemodialysis. N Engl J Med.

[14] Yeun JY, Kaysen GA (1998). Factors influencing serum albumin in dialysis patients. Am J Kidney Dis.

[15] Kaysen GA, Dubin JA, Müller HG, Rosales L, Levin NW, Mitch WE (2004). Inflammation and reduced albumin synthesis associated with stable decline in serum albumin in hemodialysis patients. Kidney Int.

[16] de Mutsert R, Grootendorst DC, Indemans F, Boeschoten EW, Krediet RT, Dekker FW (2009). Association between serum albumin and mortality in dialysis patients is partly explained by inflammation, and not by malnutrition. J Ren Nutr.

[17] Friedman AN, Fadem SZ (2010). Reassessment of albumin as a nutritional marker in kidney disease. J Am Soc Nephrol.

[18] Chantrel F, Enache I, Bouiller M, Kolb I, Kunz K, Petitjean P (1999). Abysmal prognosis of patients with type 2 diabetes entering dialysis. Nephrol Dial Transplant.

[19] Charra B, VoVan C, Marcelli D, Ruffet M, Jean G, Hurot JM (2001). Diabetes mellitus in Tassin, France: remarkable transformation in incidence and outcome of ESRD in diabetes. Adv Ren Replace Ther.

[20] Spasovski GB, Bervoets AR, Behets GJ, Ivanovski N, Sikole A, Dams G (2003). Spectrum of renal bone disease in end-stage renal failure patients not yet on dialysis. Nephrol Dial Transplant.

[21] Ferreira A, Frazão JM, Monier-Faugere M-C, Gil C, Galvao J, Oliveira C (2008). Effects of sevelamer hydrochloride and calcium carbonate on renal osteodystrophy in hemodialysis patients. J Am Soc Nephrol.

[22] Kalantar-Zadeh K (2005). editor Recent Advances in Understanding the Malnutrition-Inflammation-Cachexia Syndrome in Chronic Kidney Disease Patients: What is Next?. Semin Dial.

[23] Dukkipati R, Kovesdy CP, Colman S, Budoff MJ, Nissenson AR, Sprague SM (2010). Association of relatively low serum parathyroid hormone with malnutrition-inflammation complex and survival in maintenance hemodialysis patients. J Ren Nutr.

[24] Drew DA, Lok CE, Cohen JT, Wagner M, Tangri N, Weiner DE (2015). Vascular access choice in incident hemodialysis patients: a decision analysis. J Am Soc Nephrol.

[25] Coentrao L, Van Biesen W, Nistor I, Tordoir J, Gallieni M, Marti Monros A (2015). Preferred haemodialysis vascular access for diabetic chronic kidney disease patients: a systematic literature review. J Vasc Access.

[26] Zimmermann J, Herrlinger S, Pruy A, Metzger T, Wanner C (1999). Inflammation enhances cardiovascular risk and mortality in hemodialysis patients. Kidney Int.

[27] Brunner F, Selwood N (1992). Profile of patients on RRT in Europe and death rates due to major causes of death groups The EDTA Registration Committee. Kidney Int Suppl.

[28] Dikow R, Ritz E (2003). Cardiovascular complications in the diabetic patient with renal disease: an update in 2003. Nephrol Dial Transplant.

[29] Rodriguez J, Cleries M, Vela E (1997). Diabetic patients on renal replacement therapy: analysis of Catalan Registry data Renal Registry Committee. Nephrol Dial Transplant.

[30] Morbach S, Quante C, Ochs HR, Gaschler F, Pallast J-M, Knevels U (2001). Increased risk of lower-extremity amputation among Caucasian diabetic patients on dialysis. Diabetes Care.

[31] Ndip A, Rutter MK, Vileikyte L, Vardhan A, Asari A, Jameel M (2010). Dialysis treatment is an independent risk factor for foot ulceration in patients with diabetes and stage 4 or 5 chronic kidney disease. Diabetes Care.

[32] Khauli RB, Steinmuller DR, Novick AC, Buszta C, Goormastic M, Nakamoto S (1986). A critical look at survival of diabetics with end-stage renal disease Transplantation versus dialysis therapy. Transplantation.

[33] Mailloux LU, Bellucci AG, Napolitano B, Mossey RT, Wilkes BM, Bluestone PA (1993). Death by withdrawal from dialysis: a 20-year clinical experience. J Am Soc Nephrol.

[34] Osthus TB, von der Lippe N, Ribu L, Rustøen T, Leivestad T, Dammen T (2012). Health-related quality of life and all-cause mortality in patients with diabetes on dialysis. BMC Nephrol.

[35] Gumprecht J, Żelobowska K, Gosek K, Żywiec J, Adamski M, Grzeszczak W (2010). Quality of life among diabetic and non-diabetic patients on maintenance haemodialysis. Exp Clin Endocrinol Diabetes.

[36] Revuelta KL, López FJG, de Alvaro Moreno F, Alonso J, Group C (2004). Perceived mental health at the start of dialysis as a predictor of morbidity and mortality in patients with end-stage renal disease (CALVIDIA Study). Nephrol Dial Transplant.

[37] Hayashino Y, Fukuhara S, Akiba T, Akizawa T, Asano Y, Saito S (2009). Low health-related quality of life is associated with all-cause mortality in patients with diabetes on haemodialysis: the Japan Dialysis Outcomes and Practice Pattern Study. Diabet Med.

